# Supplementary material for: BAP1/ASXL complex modulation regulates epithelial-mesenchymal transition during trophoblast differentiation and invasion
Source: eLife. 2021 Jun 25;10:e63254. doi: 10.7554/eLife.63254 (PMC8233037; doi:10.7554/eLife.63254)
Supplement: Supplementary file 6. [file elife-63254-supp6.docx]

**Supplementary table 5: Primer sequences for RT-qPCR and CRISPR gRNAs target sequences**

| **Gene** | **Sequence** | |
| --- | --- | --- |
| **Mouse** |  | |
| *Ascl2* | F | AGCCCGATGGAGCAGGAG |
|  | R | CCGAGCAGAGGTCAGTCAGC |
| *Asxl1ex4-5* | F | GAGGAGAAGGGCTGTTTTA |
|  | R | CTCGTCTCCATCCACTGTA |
| *Asxl1 ex12* | F | AACGGGACGGACATTTTA |
|  | R | CTTGCATCTTTAGCAACCC |
| *Asxl2 ex2-4* | F | TCCAGAGAGAAGGACTGAAAG |
|  | R | GCCCATTCTACCTGGAAC |
| *Asxl2 ex11* | F | TCAAGTGATCCCAAAGCA |
|  | R | TTTACACTCGGACTGAGGAA |
| *Bap1 ex3-4* | F | TGGTGGAAGATTTCGGTGTC |
|  | R | CTGGGCTCTTCGATCCATT |
| *Bap1 ex12-13* | F | CCCTGCCATCAGATACAAGC |
|  | R | AGACATTGATGGTGTTGGGC |
| *Cdh1* | F | AGTTTACCCAGCCGGTCTTT |
|  | R | CCGGTGTCCCTATTGACAGT |
| *Cdh2* | F | GTCTGTGGAGGCTTCTGGT |
|  | R | GTCCTCGTCCACCTTGAAA |
| *Cdh15* | F | CCGAGTTCACCAAGGATGAG |
|  | R | TCACGGCTCTCATAGTCCAG |
| *mCdx2* | F | AGTGAGCTGGCTGCCACACT |
|  | R | GCTGCTGCTGCTTCTTCTTGA |
| *Ctsq* | F | AATTGGCTATGGTTATGTGGGA |
|  | R | TCACACAGTAGGGTATTGGG |
| *Egr1* | F | GAGCCGAGCGAACAACCCTA |
|  | R | TGATGGGAGGCAACCGAGTC |
| *Elf5* | F | ATTCGCTCGCAAGGTTACTCC |
|  | R | GGATGCCACAGTTCTCTTCAGG |
| *Eomes* | F | TCGCTGTGACGGCCTACCAA |
|  | R | AGGGGAATCCGTGGGAGATGGA |
| *Esrrb* | F | AGTACAAGCGACGGCTGG |
|  | R | CCTAGTAGATTCGAGACGATCTTAGTCA |
| *Gcm1* | F | ACCCCTGAAGCTTATTCCCT |
|  | R | TCGCCTTTGGACTGGAAA |
| *Hand1* | F | GAACTCAAAAAGACGGATGGTGG |
|  | R | CGCCCAGACTTGCTGAGG |
| *Id1* | F | GTCTGAAGTCGGGACCACCG |
|  | R | CTGGAACACATGCCGCCTCG |
| *Id3* | F | CAGCTGAGCTCACTCCGGAAC |
|  | R | CCAGAGTCCCAGGGTCCCAA |
| *Il17rd* | F | CGCAGTGAAAGCTGGCAGTC |
|  | R | GGCAAGGAGCTTGGAAGGGA |
| *L1cam* | F | GCAGCAAGGGTGGCAAATAC |
|  | R | TGCCAAAGGCCTTCTCTTCA |
| *Mmp2* | F | CCGAGACCGCTATGTCCAC |
|  | R | ACCACACCTTGCCATCGTT |
| *Pcdh12* | F | GCTGCTTTTGCGGAACGGAA |
|  | R | TTTGGGCTGGAATTGGCCCT |
| *Plet1* | F | CACTATGGCTAACGTCTCTGG |
|  | R | CTGTCGTCCTCCTTCACTG |
| *Prl2c2 (Plf)* | F | AACGCAGTCCGGAACGGGG |
|  | R | TGTCTAGGCAGCTGATCATGCCA |
| *Prl3b1 (Pl2)* | F | GCACTCGGGGAACAGCAGCC |
|  | R | ACTGCCAGCAACAGGAGTGCC |
| *Prl3d1 (Pl1)* | F | TTATCTTGGCCGCAGATGTGT |
|  | R | GGAGTATGGATGGAAGCAGTATGAC |
| *Prl8a9* | F | AAGAGAAAACTCCTGGAAGACC |
|  | R | AACAATTTATAATGTTTGCCCTGTG |
| *Rnf144b* | F | GCGCCCAGATGATGTGCAAGA |
|  | R | CCCCACTACCTGTGTTCGGT |
| *Sdha* | F | TGGTGAGAACAAGAAGGCATCA |
|  | R | CGCCTACAACCACAGCATCA |
| *Snai1* | F | CTTGTGTCTGCACGACCTGT |
|  | R | CTTCACATCCGAGTGGGTTT |
| *Syna* | F | CCTCACCTCCCAGGCCCCTC |
|  | R | GGCAGGGAGTTTGCCCACGA |
| *Synb* | F | TCCGGAAAGGGACCTGCCCA |
|  | R | CAGCAGTAGTGCGGGGTGCC |
| *Tfap2c* | F | GCCGGACGCCATGTTGTGGA |
|  | R | ACCCCGGTGTGCGAGAGAGG |
| *Tpbpa* | F | ACTGGAGTGCCCAGCACAGC |
|  | R | GCAGTTCAGCATCCAACTGCG |
| *Vim* | F | ACTGCAGGAGCTGAATGACC |
|  | R | CGCATCTCCTCCTCGTACAG |
| *Zeb1* | F | CATACAGAACCCAGCTTGAAC |
|  | R | CTGTGAATCCGTAAGTGCTC |
| *Zeb2* | F | GAGGAAAGAGATGGCCACG |
|  | R | GTCAGCAGTTGGGCAAAAG |
| *Zfp382* | F | TCAGACAAGGAGGCTCGT |
|  | R | CTGTAGAGGGCTTTCTGG |
| **Human** |  | |
| *AIM1* | F | TTAGTTTTAGACATTAAAGGGGG |
|  | R | TGGAAGGACCTCCAGAAGAT |
| *ASXL1* | F | GAAAAGCCACAGCCCACTAA |
|  | R | GAGCGTGAAAAGGCTGATTC |
| *ASXL2* | F | CTAAAGCAGGCGCTAAAGC |
|  | R | GCTTTGACAGTCTTTAGTGAG |
| *ASXL3* | F | CATACGTTTGCTTCCTTACCT |
|  | R | ACTTCCCATCTGCCTATCC |
| *BAP1* | F | AGGAGCTGCTGGCACTGCTGA |
|  | R | TTGTGGAGCCGGCCGATGCT |
| *CDH1* | F | GAACGCATTGCCACATACAC |
|  | R | ATTCGGGCTTGTTGTCATTC |
| *CDH2* | F | ATCCTGCTTATCCTTGTGCTG |
|  | R | GTCCTGGTCTTCTTCTCCTCC |
| *CLDN2* | F | TGGCGTCCAACTGGTGGGCT |
|  | R | ACCGCCGTCACAATGCTGGC |
| *EMP3* | F | CGAGAATGGCTGGCTGAAG |
|  | R | GCCACGCTGGTGCAAAG |
| *GAPDH* | F | CCTCAACGACCACTTTGTCAAG |
|  | R | TCTTCCTCTTGTGCTCTTGCTG |
| *GJA1* | F | AGCAGTCTGCCTTTCGTTGTAAC |
|  | R | ACCCAGAAGCGCACATGAG |
| *HLA-G* | F | CCACCACCCTGTCTTTGACTAT |
|  | R | ACGTCCTGGGTCTGGTCCT |
| *ITGA2* | F | TCACCAGGAACATGGGAACT |
|  | R | GTCAGAACACACACCCGTTG |
| *ITGB6* | F | CTACCTGTGGTGACCCCTGTAAC |
|  | R | GCTTGGCCAGCTGCTGAC |
| *PPL* | F | AGTGACCTCCTTGGTGTCGT |
|  | R | AGGGTGAATGATGGTTGGG |
| *RARRES3* | F | TGGGCCCTGTATATAGGAGATG |
|  | R | GGACTGAGAAGACACTGGAGGA |
| *SOX13* | F | AAGGATGAGCGGAGGAAGAT |
|  | R | GACTTCCAGCGAGATCCAAG |
| *TBP* | F | TGCACAGGAGCCAAGAGTGAA |
|  | R | CACATCACAGCTCCCCACCA |
| *TEAD4* | F | CGAAGGTCTGCTCTTTCGGC |
|  | R | ATGTACTCACAGAGCGGGGAC |
| *TFAP2C* | F | GAACATGCTATTGGCGGCCC |
|  | R | GTCTCCAAGACTGGGGCGAG |
| *TJP1* | F | CAACATACAGTGACGCTTCACA |
|  | R | CACTATTGACGTTTCCCCACTC |
| *VCL* | F | GGAGGTGATTAACCAGCCAAT |
|  | R | AATGATGTCATTGCCCTTGC |
| *YWZA* | F | ACTTTTGGTACATTGTGGCTTCAA |
|  | R | CCG CCAGGACAAACCAGTAT |
| *ZEB2* | F | AAGCCAGGGACAGATCAGC |
|  | R | CCACACTCTGTGCATTTGAACT |

**PCR screening primers**

| *Asxl1* | F | AACTGGTTTGGGAGTTCACG |
| --- | --- | --- |
|  | R | TGTCTGCCACAGGGTTTCT |
| *Asxl2* | F | GTCTCTGAGGACACGTGCAA |
|  | R | CCAAGACCACCACATCACAG |

**CRISPR gRNAs target sequences**

| *Asxl1* (exon 4) | Upstream | AGAGCTTGGTTCGTATTGTC |
| --- | --- | --- |
|  |  | CCCACTCAGAGTCTAGGTTG |
|  | Downstream | AGACATTAGTGATACCTGAC |
| *Asxl2* (exon 2) | Upstream | CACAGATAGGGATAGGACTT |
|  |  | ATCAGTAACTACTACTGAAT |
|  | Downstream | TTACTAGTAATGATTGTGTA |
|  |  | ATATTGAGGTTGGTAATTAT |
| *Bap1* SAM gRNA1 |  | CCGCCTCCGCCCCCGCCGTT |
| *Bap1* SAM gRNA2 |  | ATGCACGCGCGCGCGCGTCG |
| *Bap1* SAM gRNA3 |  | GCGAGGGCGCGCACGTGCGG |
| Non-targeting*-1* |  | GCTTTCACGGAGGTTCGACG |
| Non-targeting*-2* |  | ATGTTGCAGTTCGGCTCGAT |
